# Eco-Physiological Screening of Different Tomato Genotypes in Response to High Temperatures: A Combined Field-to-Laboratory Approach

**DOI:** 10.3390/plants9040508

**Published:** 2020-04-15

**Authors:** Carmen Arena, Stefano Conti, Silvana Francesca, Giuseppe Melchionna, Josef Hájek, Miloš Barták, Amalia Barone, Maria Manuela Rigano

**Affiliations:** 1 Department of Biology, University of Naples “Federico II”, Complesso Universitario Monte S. Angelo, Via Cintia, 80126 Napoli, Italy; c.arena@unina.it; 2 Department of Agricultural Sciences, University of Naples “Federico II”, Via Università 100, 80055 Portici (NA), Italy; stefano.conti@unina.it (S.C.); silvana.francesca@unina.it (S.F.); giuseppe.melchionna@unina.it (G.M.); ambarone@unina.it (A.B.); 3 Department of Experimental Biology, Faculty of Science, Masaryk University, University Campus Bohunice, Kamenice 753/5, 62500 Brno, Czech Republic; jhajek@sci.muni.cz (J.H.); mbartak@sci.muni.cz (M.B.)

**Keywords:** heat stress, tomato genotypes, photosynthesis, crop yield, chlorophyll a fluorescence, *Solanum lycopersicum*

## Abstract

High temperatures represent a limitation for growth and development of many crop species. Several studies have demonstrated that the yield reduction of tomato under high temperatures and drought is mainly due to a photosynthetic decline. In this paper, a set of 15 tomato genotypes were screened for tolerance to elevated temperatures by cultivating plants under plastic walk-in tunnels. To assess the potential tolerance of tomato genotypes to high temperatures, measurements of chlorophyll fluorescence, pigments content and leaf functional traits have been carried out together with the evaluation of the final yields. Based on the greenhouse trials, a group of eight putative heat-sensitive and heat-tolerant tomato genotypes was selected for laboratory experiments aimed at investigating the effects of short-term high temperatures treatments in controlled conditions. The chlorophyll fluorescence induction kinetics were recorded on detached leaves treated for 60 min at 35 °C or at 45 °C. The last treatment significantly affected the photosystem II (PSII) photochemical efficiency (namely maximum PSII quantum efficiency, F_v_/F_m,_ and quantum yield of PSII electron transport, Φ_PSII_) and the non-photochemical quenching (NPQ) in the majority of genotypes. The short-term heat shock treatments also led to significant differences in the shape of the slow Kautsky kinetics and its significant time points (chlorophyll fluorescence levels minimum O, peak P, semi-steady state S, maximum M, terminal steady state T) compared to the control, demonstrating heat shock-induced changes in PSII functionality. Genotypes potentially tolerant to high temperatures have been identified. Our findings support the idea that chlorophyll fluorescence parameters (i.e., Φ_PSII_ or NPQ) and some leaf functional traits may be used as a tool to detect high temperatures-tolerant tomato cultivars.

## 1. Introduction

Increasing atmospheric temperatures, which are expected to rise by 2–4.8 °C in the next few decades, can compromise crop productivity in numerous regions worldwide [1,2,3,4]. Indeed, elevated temperatures can induce a series of physiological responses with consequent decreases of crops yields and quality [5,6]. Tomato (*Solanum lycopersicum*), being an excellent source of health-promoting compounds, is one of the most important crops cultivated worldwide and its heat sensitivity varies among different genotypes [4,7,8]. Generally, the optimal temperature range for photosynthesis is considered to be between 25 °C and 30 °C [6]. The rising of average temperatures due to the ongoing climate change will cause extensive productivity losses in Mediterranean areas, where tomato is traditionally cultivated [9,10,11]. In this framework, it becomes important to perform studies that are able to identify the most promising genotypes able to face heat stress.

The relationship between gas exchange and crop yield has been largely studied in tomato, suggesting leaf transpiration as the most reliable indicator for yield prediction under drought [12]. However, beside gas exchange, other photosynthesis related parameters [13] should be taken into account to build a “eco-physiological identity card” for different genotypes.

Chlorophyll fluorescence represents a good tool to rapidly and accurately detect plant health status, the occurrence of damage within photosystem II (PSII), and to study heat tolerance in vivo [6,13,14]. The decline of maximum quantum efficiency of PSII (F_v_/F_m_), as well as the increase of non-photochemical quenching (NPQ), are two heat-affected fluorescence parameters [15] related to photoinhibition and photoprotection mechanisms in response to high temperatures [5,10,16]. Also, transient changes in fluorescence intensity (Kautsky phenomenon) have been demonstrated to be particularly suitable for the screening of physiological parameters in plants. The shape of fluorescence curves changes significantly when plants switch from a healthy status to stress, giving precious information on plant capability to overcome the stress [17].

Indeed, physiological screening techniques may complement phenotypic measurements and, therefore, increase the efficiency of the selection of tolerant genotypes [18]. Currently, the majority of the experiments on tomato responses to heat stress have been carried out in controlled chambers and only few studies have been performed in the field [11,14,19].

On one hand, with the field approach, it is possible to screen a high number of different genotypes. On the other hand, it is not easy to separate the effects of heat stress from other environmental variables, such as light or water depletion, in inducing the plant specific responses being examined [18]. For these reasons, field experiments should be complemented by laboratory studies in which the use of novel screening methods, such as chlorophyll fluorescence imaging, may provide further information for the characterization of tomato genotypes best suited to different environmental conditions.

This study aimed to evaluate the responses to elevated temperatures in tomato genotypes in a combined field-to-laboratory approach. First, we investigated the photosynthetic efficiency by fluorescence emission measurements and leaf structural traits of different tomato varieties grown in Mediterranean agro-ecosystems during summer in the field. These trials allowed us to identify functional parameters correlating with final crop yields in different genotypes. After, a group of heat-tolerant and heat-sensitive tomato genotypes were selected based on crop yield and photosynthesis-related parameters. These selected genotypes were further characterized in the laboratory, analyzing the Kautsky fluorescence induction curve after short-term heat treatments to obtain “a signature of photosynthesis”. More specifically, the shapes of the curves changed when plants were subjected to stress. The presence and the timing of the appearance of specific fluorescence transients (for definitions, see [20]) were calculated in order to assess the heat stress-induced changes in PSII functionality among cultivars.

These laboratory analyses allowed us to validate this easy and very quick protocol as an alternative method for the selection of potentially heat-tolerant tomato genotypes. The combined field-to-laboratory approach provides additional information which could help plant biologists and breeders with characterizing responses to high temperatures in *Solanum lycopersicum*.

## 2. Results

### 2.1. Greenhouse Trials: Correlations Between Physiological Parameters and Yields

Fifteen tomato genotypes, differing in geographical origin, were grown at supra-optimal temperatures in order to identify the high and low performers in field environmental conditions. The genotypes were selected on the basis of crop yield and eco-physiological indices. During anthesis, maximum air temperatures inside the greenhouse were in the range of 24–43 °C. Significant differences between each genotype vs. the control genotype JAG8810 were recorded for leaf functional traits, chlorophylls and carotenoids contents and final yields, as reported in Table 1. The hybrid JAG8810 had a good yield under high temperatures (from Monsanto, unpublished results). Therefore, this line may be considered as a positive control which is able to resist extreme high temperatures.

**Figure 1:**
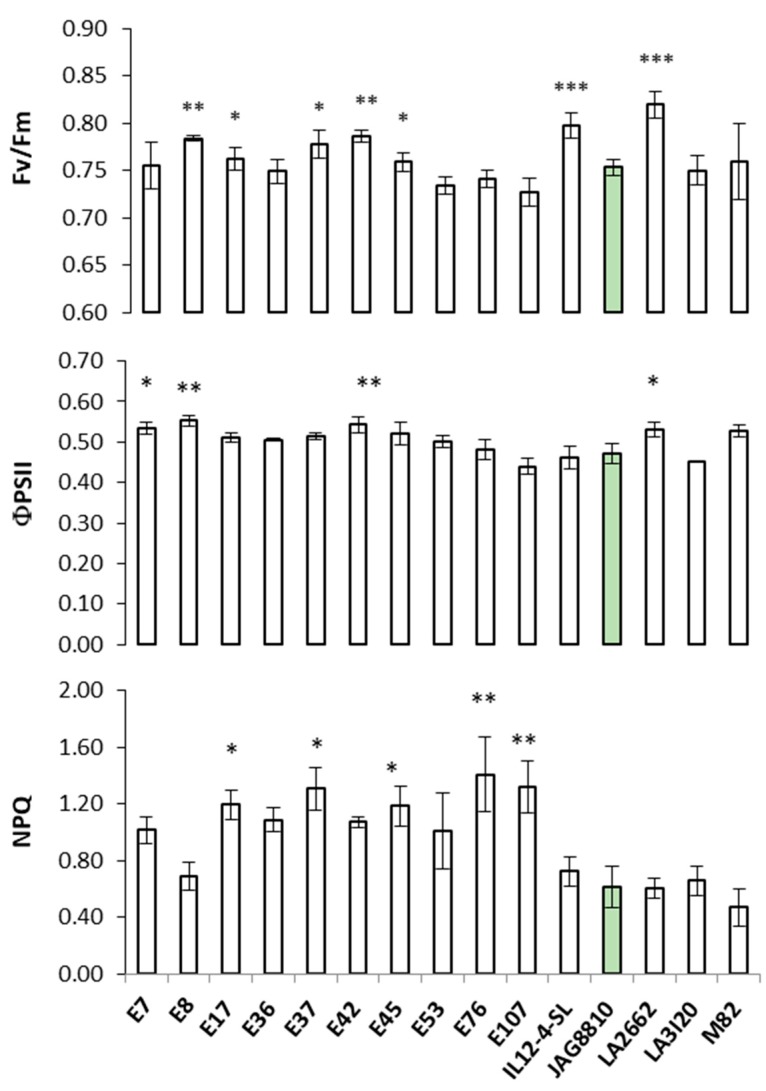
Maximum quantum yield of photosystem II (PSII) (F_v_/F_m_), effective quantum yield of PSII (Φ_PSII_), and non-photochemical quenching (NPQ) measured on different tomato genotypes grown under a plastic walk-in tunnel. Bars are means ± standard error (*n* = 5). ANOVA with LSD post-hoc test was used to compare each genotype vs. the control genotype JAG8810 (green column). The asterisks indicate statistically significant differences at * *p* < 0.05; ** *p* < 0.01; *** *p* < 0.001.

Leaf dry weight (DW) and leaf area (LA) of genotypes E36, E45, E76, LA2662, LA3120 and M82 were significantly higher compared to JAG8810. The genotype E7 was the only one with SLA values higher than JAG8810. The pigments content (chlorophyll a and b and carotenoids) of all genotypes was significantly lower than JAG8810, whereas only the M82 genotype showed a crop yield higher than JAG8810, considered as the control. Chlorophyll fluorescence parameters (F_v_/F_m_, Φ_PSII_ and NPQ) are shown in Figure 1.

Based on field screening, the photochemical parameters differed among genotypes; more specifically, seven genotypes, namely E8, E17, E32, E37, E42, IL12-4-SL and LA2662, showed higher F_v_/F_m_ values compared to JAG8810. The genotypes E8, E42 and LA2662 also exhibited higher Φ_PSII_ compared to the control genotype. Conversely, E76 and E107 genotypes showed the highest NPQ values compared to the control and the other genotypes. The correlations among the analyzed physiological and structural parameters are reported in Table 2.

Crop yield was negatively correlated with NPQ. Leaf area was positively correlated with leaf DW. The maximum quantum efficiency of PSII (F_v_/F_m_) differed among genotypes, although no significant correlation was found between crop yield and F_v_/F_m_. No correlations were found between crop yield and chlorophylls or carotenoids content.

Based on crop yield (YP) and NPQ (higher YP and lower NPQ, Table 1 and Figure 1), the top five performers (genotypes IL12-4-SL, JAG8810, LA3120, LA2662 and M82) along with the low performer (genotype E107), were chosen for further analyses.

### 2.2. Chlorophyll a Fluorescence Measurements on Detached Leaves: Heat Shock Treatment at 35 °C and 45 °C

Chlorophyll fluorescence transients and derived parameters were measured as described in the Materials and Methods section on detached leaves from the selected genotypes and from an additional two tomato varieties, BG1620 and E41, which are supposedly heat-sensitive from previous studies carried out in our laboratory.

No significant differences in the chlorophyll fluorescence parameters after a 60-min heat shock at 35 °C were found between control and treated leaves (data not shown).

**Figure 2:**
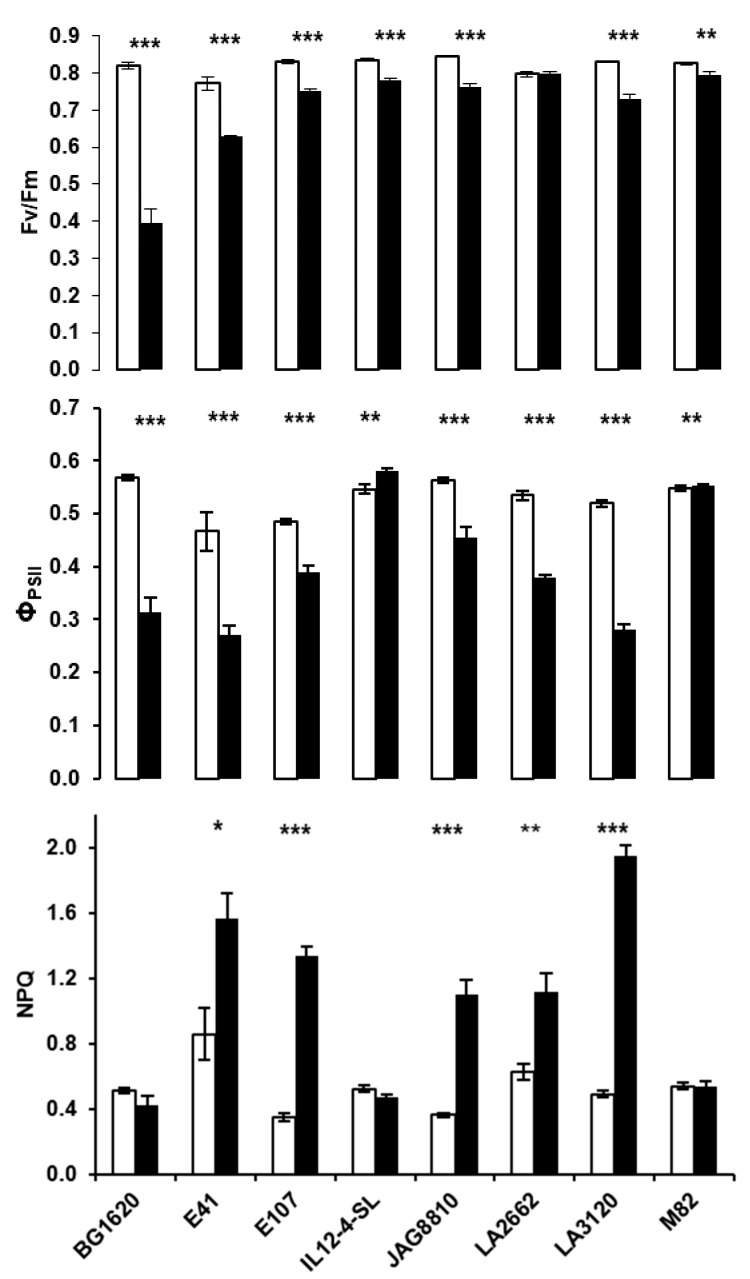
Maximum quantum yield of PSII (F_v_/F_m_), effective quantum yield of PSII (Φ_PSII_) and non-photochemical quenching (NPQ) in detached tomato leaves of different tomato genotypes following short term heat treatment for 60 min at 45 °C (solid bars), compared with the respective non-treated control (open bars). Bars are means ± standard error (*n* = 5). The asterisks indicate statistically significant differences (* *p* < 0.05; ** *p* < 0.01; *** *p* < 0.001) according to Student’s t-test.

Conversely, heat shock at 45 °C for 60 min resulted in significant damage to PSII compared to control (Figure 2).

The short-term heat shock treatment led to a reduction in maximum PSII quantum efficiency (F_v_/F_m_) in almost all genotypes. Interestingly, the highest reductions in F_v_/F_m_ were recorded in the two supposedly heat-sensitive genotypes, BG1620 (−52%) and E41 (−19%), that were more affected by heat stress compared to the genotype LA3120 (−12% F_v_/F_m_ reduction). Contrastingly, the F_v_/F_m_ ratio was little or not affected by the heat shock treatments in M82 (−4%) and LA2662 (0%) genotypes, which were among the top performers in the field trial. The quantum efficiency of PSII electron transport (Φ_PSII_) was also affected by the heat shock treatment. For most genotypes, a significant reduction in the Φ_PSII_ was recorded and LA3120, BG1620, and E41 were found to be the most sensitive genotypes with a decrease of −46%, −45% and −42% compared to the control, respectively. The M82 and IL12-4-SL genotypes were little or not affected by the heat shock treatment. As a consequence of the heat shock treatment, the NPQ values increased significantly in most genotypes compared to their respective controls, but not in BG1620, IL12-4-SL and M82.

### 2.3. Heat-Induced Changes in Shape of Kautsky Kinetics

**Figure 3:**
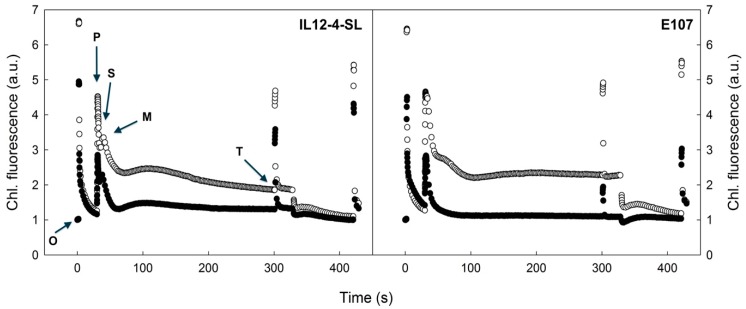
The effect of heat shock (60 min at 45 °C) on the shape of slow Kautsky kinetics in tomato plants. Non-treated controls are indicated by open symbols (◯), heat shock-treated leaves by solid symbols (●). Less sensitive (IL12-4-SL) and substantially sensitive genotypes (E107) are presented. The curves are means of at least 4 replicates (leaves). Data are normalized to background chlorophyll fluorescence (F_0_). The chlorophyll fluorescence levels: O, fluorescence minimum; P, fluorescence peak; S, semi-steady state; M, fluorescence maximum; T, terminal steady state are indicated.

The shape of slow Kautsky kinetics and the derived parameters clearly showed that the effects of heat treatment vary with genotype. As an example, in Figure 3, the slow Kautsky kinetics of the least heat sensitive (IL12-4-SL) and the most heat sensitive (E107) genotype are reported. Heat treatment leads to a reduction of the P peak in both genotypes compared to non-stressed controls. This decline appears more pronounced for the heat sensitive genotype E107. The comparison among genotypes showed that the M and S chlorophyll fluorescence signals were missing in heat-treated samples and resulted in the absence of P/S, P/M, and S/M ratios compared to respective controls (Table 3). 

It may be generalized that for all tomato genotypes the shape of the slow Kautsky kinetics was affected mainly during the early phase (i.e., within the first 60 sec after the actinic light was switched on). The most pronounced change was found in the O/P ratio in BG1620 which showed a relative change of about 200%, while other genotypes showed a much lower variation (LA2662: 5.4%). Apart from the shape of the slow Kautsky kinetics and ratio values, the time at which the P, S, and M points were reached differed within treatments. Heat treatment led to an increase in *t_P_* in four cultivars. 

However, *t_P_* showed no change in three genotypes, and a decrease in the IL12-4-SL genotype.

## 3. Discussion

To date, there is a lack of knowledge about how differently sensitive/tolerant tomato genotypes would respond to heat events. In heat-sensitive tomato genotypes, high temperatures are responsible for the decrease in photosynthesis and overall crop yield [6].

In this paper, a set of eco-physiological parameters have been proposed to screen the most promising tomato genotypes to be cultivated under elevated temperatures. A combined field/laboratory experimental approach was performed. Firstly, a field trial was carried out under a plastic walk-in tunnel to assess the field performances of tomato genotypes cultivated at high temperatures (up to 43 °C) in terms of crop yield and physiological traits. In the second part of this study, the heat-resistant and heat-sensitive genotypes were tested in the laboratory to analyze their responses to short-term heat shock and to investigate the photochemical behavior related to their different field performance.

We suppose that at the temperatures considered in this study, the photosynthetic apparatus was not damaged but rather regulated in the different genotypes, contributing to the degree of their sensitivity or resistance to heat.

Our field studies indicated that tomato genotypes with higher yields also had lower NPQ values. Non-photochemical quenching is considered a key mechanism in photoprotection against light and temperature stress in higher plants [16,21]. High NPQ is often associated with conformational changes within PSII, which transiently depresses CO_2_ fixation [22].

In our trials, the top performers in terms of yield (genotypes IL12-4-SL, M82, LA2662, LA3120 and JAG8810) also showed the lowest NPQ values. Our data suggest that under experimental field conditions, these tomato genotypes are more efficient in transferring the light excitation energy to CO_2_ fixation, thus producing a higher photosynthetic carbon gain. Contrastingly, the highest NPQ values measured in the low performing genotypes (E37, E76 and E107) correspond to more intense thermal energy dissipation, leading to lower CO_2_ assimilation and crop yield.

In heat-tolerant tomato genotypes, the NPQ protection is likely activated less promptly than in the heat-sensitive genotypes. Conversely to NPQ, the F_v_/F_m_ ratio and Φ_PSII_ did not show any correlation with crop productivity, suggesting that the higher F_v_/F_m_ values in some genotypes may indicate a better photosynthetic performance that is not always related to a higher crop yield. These data are in contrast with findings of many authors, who demonstrated that the F_v_/F_m_ ratio is one of the fluorescence parameters most affected by high temperatures and used it as an index for screening tomato genotypes under heat stress [14,15,23]. Indeed, our data indicated that the high temperatures experienced by plants in the field did not compromise the quantum yield of PSII in any genotype.

**Figure 4:**
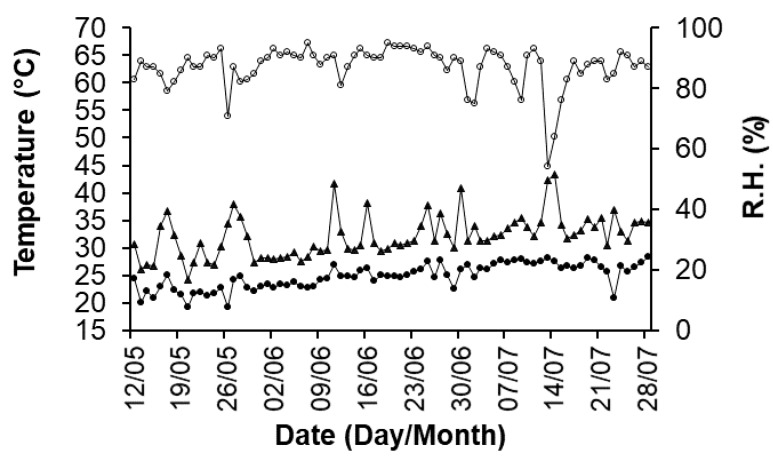
Relative humidity (R.H.; open symbols, right axis) and maximum and minimum air temperatures (solid symbols, left axis) during May–July 2017 inside a greenhouse in the experimental field at Battipaglia (Salerno, Campania region, Italy).

Based on the results of Hückstädt et al. [24], it may be hypothesized that the detrimental effect of the high diurnal temperatures on photosystems has been compensated for by the optimal temperatures [6] reached in the greenhouse during the night (see Figure 4).

Elevated temperatures could also determine a loss in the amount of photosynthetic pigments. Therefore, the capacity of some genotypes to maintain higher pigments content under heat stress, as well as to adjust some leaf functional traits (i.e., SLA, LA, RWC), are considered key heat tolerance-linked traits [6,25,26,27].

For example, a higher SLA can be essential to obtain a higher potential evaporative demand and a more extensive foliar display to capture more light [26]. However, in this work, no significant correlation was found between final crop yield and pigments content or final crop yield and SLA, indicating that these traits are not automatically linked to crop productivity. Therefore, these traits were not considered for the selection of the heat-tolerant and heat-sensitive genotypes in this study.

On the basis of field trials, the tomato genotypes IL12-4-SL, JAG 8810, LA3120, LA2662 and M82 were selected as the top performers in terms of yield and low NPQ values, whereas the genotype E107 was selected as a low performer considering the low photochemical efficiency and crop yield. The heat sensitive BG1620 and E41 genotypes were added to the list of genotypes to be further studied in the laboratory. For these tomato genotypes, the chlorophyll fluorescence transient analysis (slow Kautsky kinetics) was performed in response to heat treatments to separate the effects of the high temperature from other environmental constraints in the field.

In a previous study on tomato, plants exposed to 42 °C for 24 h showed a decline of net photosynthesis, maximum PSII photochemical efficiency and electron transport rate [28]. Consistent with these findings, our results further demonstrated that a short-term (60 min) heat shock at 45 °C was sufficient to cause significant effects on the photochemistry in detached tomato leaves. Interestingly, the F_v_/F_m_ ratio was found to be most affected in the heat-sensitive genotype BG1620 and in the low performer E107 genotype. An F_v_/F_m_ reduction of only 8% was registered in the genotype LA2662, which was selected as heat-tolerant in the field experiment. These data are also in agreement with Sharma et al. [18] who measured a reduction of F_v_/F_m_ in detached wheat leaves at 45 °C and Camejo et al. [29] who reported an F_v_/F_m_ decrease in a heat-susceptible tomato cultivar and no changes in heat-tolerant cultivar.

We supposed that the F_v_/F_m_ and PSII decreases in the sensitive (BG1620 and E41) genotypes could be due to heat-induced structural modifications in PSII, particularly D1 protein oxidative degradation. It has been demonstrated that heat stress may cause cleavage of the reaction center-binding protein D1 and induce dissociation of a manganese-stabilizing 33 kDa protein from the PSII reaction center complex [30]. Such oxidative damages have a strong positive relationship with the accumulated levels of reactive oxygen species (ROS) and lipid peroxidation under heat stress [31].

However, it cannot be excluded that the significant reduction of F_v_/F_m_ observed in some genotypes may also be associated with photoprotection mechanisms, as indicated by NPQ values that peaked in correspondence to the reduction in F_v_/F_m_. Simultaneously, protective mechanisms are activated to protect the D1 protein, such as the expression of heat shock proteins (HSPs) like HSP21 that directly binds D1 to shield it against damage [32]. However, this does not seem to be the case for the genotype BG1620, which was the most affected by the severe decline of photochemical and non-photochemical processes.

The decrease in F_v_/F_m_ values in detached tomato leaves that were heat-treated at 40 °C was also reported by Willits and Peet [33]. In our work, a heat shock treatment at 35 °C on detached leaves was similarly tested with no significant effects on the photochemical efficiency of PSII (data not shown), supporting the idea that such responses depend on the severity of the heat stress applied [18].

The analysis of the shape of the slow Kautsky kinetics and the parameters calculated from its significant time points (chlorophyll fluorescence levels O, P, S, M, T) revealed that heat shock treatment led to significant differences compared with the control. Polyphasic changes in chlorophyll fluorescence signal in the PSMT part of the Kautsky kinetics represent the combined effect of photochemical and non-photochemical processes taking place in the chloroplast [34]. As the main changes happened during the first 60 s of actinic light exposure, they might be attributed to the interperiod of balancing the rate of primary photochemical processes in PSII to the rate of CO_2_ assimilation.

Since the S and M chlorophyll fluorescence levels were generally missing in heat-treated tomato leaves, the processes responsible for S and M interstates (i.e., the processes regulating the Calvin–Benson cycle of CO_2_ fixation, such as limitations in NADP+, phosphate pool equilibration, and transmembrane ΔpH formation [20]) were overwhelmed by a strong non-photochemical quenching that was activated by the heat shock treatment. This may be a consequence of the heat-induced thermal dissipation of absorbed light energy, such as state 1 to state 2 transitions causing preferential excitation of PSI and structural changes in thylakoid membranes, as reported by Marutani et al. [35]. 

The activation of these protective mechanisms may, however, lead to PSI damage in high temperature treated plants due to over-reduction of the acceptor side of PSI [13].

The changes observed during the transition from P to S phase of the Kautsky kinetics indicate the actual proportion between the mechanisms involved in photochemical and non-photochemical quenching [36]. Since the parameters derived from the slow Kautsky kinetics responded to heat treatment, it might be concluded that they have a high potential in the evaluation of heat effects on the chloroplast function of tomato, as shown for light stress and leaf age effects by Nesterenko et al. [37]. Many studies support the idea that a sustained increase in leaf photosynthesis can also lead to an increase in total biomass production [38].

Overall, in this work, several useful photosynthetic parameters were identified, which could be essential to detect and describe high temperature-tolerant tomato cultivars. These parameters could be used as an effective tool for the prompt identification of tomato genotypes tolerant to high temperatures.

## 4. Materials and Methods

### 4.1. Plant Material and Growth Conditions

Fifteen genotypes have been tested in the field. Of these genotypes, eleven genotypes (marked as E## in Table 4) were selected based on their different productivity (demonstrated in a previous experiment conducted in the Campania region in the year 2016 [39]). The genotypes JAG8810 (Monsanto F_1_ hybrid), LA2662 (Saladette) and LA3120 (Malintka) were reported to have high fruit productivity under high temperatures (JAG8810, from Monsanto, unpublished results; LA2662 and LA3120, Tomato Genetic Resources Center [40]). The JAG8810 hybrid may be considered as a positive control which is able to resist extreme high temperatures. The M82 and the IL12-4-SL genotypes, previously selected and characterized in a recent paper [41], were added to the list of genotypes to be tested because their physiological response to elevated temperatures was unknown. One additional tomato variety, BG1620 (kindly provided by Prof. G. Pevicharova, MVRCI Bulgary), was also added to the set of genotypes to be analyzed. The genotypes BG1620 and E41 are supposedly heat-sensitive based on previous analyses carried out in our laboratories (unpublished data).

Tomato plants were grown in the year 2017 in Battipaglia (Salerno, Italy) (40°23′03 N, 17°21′17 E, 72 m a.s.l.) in a Mediterranean or Csa climate according to the Köppen classification scheme [42], under walk-in thermal polyethylene tunnels. During the whole cultural cycle, climatic data were recorded using the weather station VantagePro2 from Davis Instrument Corp. The maximum and minimum temperatures during anthesis are reported in Figure 4. Spatial variation in temperature within the walk-in tunnels was found to be minimal.

The seeds of all genotypes were first rinsed and soaked in distilled water and then kept for 4 days in 8.5 cm diameter Petri dishes over 3 layers of filter paper saturated with distilled water. After germination, the seeds were sown in seed trays kept in the greenhouse. Seedlings were transplanted in April under plastic walk-in tunnels. Plants were grown following the standard cultural practices of the area. Insecticides and fungicides were applied to the plants according to general local practices and recommendations. Urea phosphate fertilizer (40 kg ha^−1^) was applied to the soil before transplanting. Tillage treatments included plowing that was followed by one/two milling. Weeding and ridging were also carried out. Through fertirrigation, recommended levels of N (190 kg ha^−1^) and K (20 kg ha^−1^) were applied. During cultivation, plants were irrigated as required. 

All genotypes were grown according to a completely randomized experimental design with three replicates and 10 plants *per* replicate. Total fruit number and fresh weight were measured at the end of growth season to evaluate the crop yield *per* plant (YP). The crop yield was measured at the red fruit ripe stage.

### 4.2. Functional Leaf Trait Analysis

The measurements of leaf area (LA), specific leaf area (SLA) and leaf dry weight (DW) were performed on the fourth leaf from the apex in each plant. Five leaves for each genotype were sampled from 5 different plants. LA was measured using ImageJ 1.45 software for image analysis [43]. The leaves were then dried at 70 °C and their DW was measured after 48 h. SLA was calculated as the ratio of leaf area to leaf dry weight and expressed as cm^2^ g^−1^ DW according to Cornelissen et al. [44].

### 4.3. Chlorophyll Fluorescence Emission Measurements in the Field

Chlorophyll fluorescence parameters were measured on fully expanded leaves (the fourth leaf from the apex) using a portable FluorPen FP100 Max fluorometer, equipped with a Photosynthetically Active Radiation (PAR) sensor (Photon Systems Instruments, Drásov, Czech Republic) following the procedure reported by Sorrentino et al. [45]. Five replicate measurements for each genotype were taken as follows.

The ground state fluorescence (F_0_) was induced by an internal Light Emitting Diode (LED) blue (1–2 μmol photons m^−2^ s^−1^) on 30-min dark-adapted leaves of plants moved into a dark room. The maximum fluorescence level in the dark-adapted state (F_m_), was triggered by a 1 s saturating light pulse of 3000 μmol photons m^−2^ s^−1^, and the maximum quantum efficiency of PSII (F_v_/F_m_) was calculated according to Equation (1).

The fluorescence readings in the light were taken using an open leaf-clip, allowing for measurements of the steady state fluorescence level (Fs) at an ambient light Photosynthetic Photon Flux Density (PPFD) of 150–200 µmol m^−2^ s^−1^ in the PAR spectrum and of the maximum fluorescence level in light-adapted leaves (F’_m_) measured after a saturating light pulse.

The quantum yield of PSII electron transport (Φ_PSII_) was calculated according to Equation (2) [46]. Non-photochemical quenching (NPQ) was calculated using Equation (3) [47].

F_v_/F_m_ = (F_m_ − F_0_)/F_m_ (1)

Φ_PSII_ = (F’_m_ − F_s_)/F’_m_ (2)

NPQ = (F_m_ − F’_m_)/F’_m_ (3)

### 4.4. Determination of Total Chlorophylls and Carotenoids Content

Following the chlorophyll fluorescence field measurements, the same leaves were excised, stored in a cool box and transferred to the laboratory for the determination of photosynthetic pigments content (chlorophyll a, chlorophyll b and carotenoids) according to the method described by Rigano et al. [5]. One gram of leaf sample was extracted with 16 mL of acetone/hexane (40/60, *v*/*v*) with a T-25 Ultra-Turrax Homogenizer (IKA-Werke GmbH & Co. KG, Staufen, Germany). The homogenate was then centrifuged at 5000 rpm for 5 min at 4 °C and supernatants were collected and stored at −20 °C prior to spectrophotometric analysis. Pigment contents were calculated in mg on 100 g of leaf fresh weight. Three separate biological replicates for each sample and three technical assays for each biological repetition were measured.

### 4.5. Chlorophyll a Fluorescence—Heat Treatments and Laboratory Measurements

Chlorophyll fluorescence transients and numeric parameters were measured with a FluorCam (Photon Systems Instruments, Brno, Czech Republic) on detached leaves from genotypes selected from the in-field analyses. Two additional tomato varieties, BG1620 and E41 (supposedly heat-sensitive based on previous analyses carried out in our laboratories (unpublished data), were also added to the set of genotypes to be studied in this research.

Whole compound leaves from field grown tomato plants were sampled in the morning, between 2 and 3 h after dawn (8:00 to 9:00 a.m.). Fully expanded leaves (the fourth leaf from the apex) were excised at the base of the petiole from each plant using a sharp blade and the cut base was immediately immersed in distilled water in a 50 mL test tube in order to prevent dehydration. The sampled leaves were then temporarily stored in a dark cool box and transferred to the laboratory for the short-term high temperature treatments and chlorophyll fluorescence experiments.

In the laboratory, single leaflets were excised from the tomato compound leaves and placed in 9.0 cm diameter Petri dishes over water saturated filter paper. Two groups of leaf samples were selected for each genotype: one group was kept at the laboratory room temperature (25–26°C) in the dark and assumed as control treatment, while another group was placed in a thermostatic cabinet set, either at 35°C or at 45°C, for 1 h in the dark and was considered the heat treatment. At the end of the heat treatment, leaf samples were adapted at room temperature for 20 min in the dark prior to performing the fluorescence measurements. The same procedure was replicated on different leaves and repeated for each tomato genotype. The whole experiment was carried out subsequently on leaf samples treated either at 35 °C (lower heat stress level) or at 45 °C (higher heat stress level), plus the respective controls.

The temperature conditions in this experiment were chosen to represent the optimal temperature range for tomato (within 25–30 °C [6]) and the maximum air temperature of 33–43 °C encountered by tomato genotypes in the walk-in tunnels during the experimental period. Chlorophyll fluorescence transients (slow Kautsky kinetics supplemented with quenching analysis) were measured with a Handy Fluor Cam FC-1000H imaging fluorimeter (Photon Systems Instruments, Drásov, Czech Republic) controlled by the FluorCam7 software (Photon Systems Instruments, Drásov, Czech Republic).

The experimental protocol started with the measurement of the ground state (minimum) fluorescence level (F_0_, O) when the samples were exposed to low intensity measuring light flashes, followed by a saturating pulse of light (960 ms, 2400 μmol m^−2^ s^−1^) to induce maximum chlorophyll fluorescence (F_m_, P). After 27 s of dark adaptation, the samples were exposed to actinic light (200 μmol m^−2^ s^−1^) for 5 min until steady state chlorophyll fluorescence (F_s_) was reached. At this point, another saturating pulse of light induced the maximum chlorophyll fluorescence of the light-adapted sample (F’_m_). The maximum quantum efficiency of PSII (F_v_/F_m_), the quantum efficiency of PSII electron transport (Φ_PSII_) and non-photochemical quenching (NPQ) were calculated using the FluorCam7 software, according to Equations (1)–(3) reported above, respectively.

### 4.6. Analysis of Kautsky Kinetics Shape in Response to Heat Treatment

Slow Kautsky kinetics is routinely used to evaluate the sensitivity of plants to a wide variety of stressors [48,49]. In this study, the analysis of kinetic fluorescence shape, i.e., the presence and the time of appearance of specific fluorescent transients (O, P, S, M and T), was utilized to assess heat stress-induced changes in PSII functionality in tomato. For individual Kautsky kinetics recorded after a heat treatment (see above), chlorophyll fluorescence levels O, P, S, M and T were identified, as well as the times at which they were reached. Effects of experimental temperature on the O-, P-, S-, M-, and T-derived parameters were then evaluated for the individual genotypes and the genotype-dependent responses of the parameters were characterized.

### 4.7. Statistical Analysis

Statistical analysis was performed on all measured traits using SPSS 23 Software (IBM SPSS Statistics, USA). Analysis of variance (ANOVA) was used to check for significant differences between each genotype vs. the control genotype (JAG8810) and where significant differences were found, the least significant difference (LSD) at the 0.05, 0.01 or 0.001 level of probability was calculated and used to compare the mean values. Student’s t-test was performed to check for differences between control and heat-treated samples in the case of detached leaf experiments. Pearson’s correlation coefficient was used to test associations between tomato yield and other variables.

## 5. Conclusions

Due to ongoing climate change, the screening and identification of the most promising tomato cultivars able to maintain elevated productivity under heat stress becomes a priority for farmers and producers to avoid significant losses of crop yield.

In our experiments, heat tolerant and heat sensitive tomato cultivars were identified and characterized using a correlative approach combining different field and laboratory methods based on functional leaf traits, crop yield and photochemical indexes.

The three main outcomes emerging from this work include the confirmation that some parameters linked to chlorophyll fluorescence emission can be used to phenotype heat tolerance in tomato, both in the field and in the laboratory. Secondly, we demonstrated that the detached leaf method can be used as an easy, quick and valid alternative for the selection and characterization of potentially heat-tolerant tomato genotypes. The advantage of a laboratory approach that implements field measurements is that it is possible to separate the effects caused by heat treatments from the other related environmental factors such as high light, low relative humidity and limited water supply. Finally, we identified five tomato genotypes (JAG8810, LA3120, LA2662, IL12-4-SL and M82) as promising genotypes that are potentially tolerant to elevated temperatures. These genotypes also represent a valuable resource to be used in future works aiming to assess the underlying physiological mechanisms for variability in photosynthetic responses among different cultivars.

## Figures and Tables

**Table 1:** Leaf traits (DW = dry weight of individual leaves, LA = leaf area, SLA = specific leaf area), total carotenoids, chlorophylls (Chl a and Chl b) and crop yield *per* plant (YP) evaluated on tomato genotypes grown under a plastic walk-in tunnel. Values are means ± standard deviation. ANOVA with Least Significant Difference (LSD) post-hoc test was used to compare each genotype vs. the control genotype JAG8810. The asterisks indicate statistically significant differences at * *p* < 0.05; ** *p* < 0.01; *** *p* < 0.001.

Genotypes	DW (g)	LA (cm^2^)	SLA (cm^2^ g^−1^)	Carotenoids (mg 100 g^−1^)	Chl a (mg 100 g^−1^)	Chl b (mg 100 g^−1^)	YP (kg pt^−1^)
E7	0.04 ± 0.10	11.41 ± 1.71	278.17 ± 63.62 *	28.76 ± 0.07 ***	104.82 ± 0.13 ***	36.53 ± 0.29 ***	0.98 ± 0.00 **
E8	0.10 ± 0.04	14.37 ± 2.86	155.73 ± 36.29	41.30 ± 0.25 ***	169.30 ± 0.27 ***	76.05 ± 0.46 ***	0.77 ± 0.17 ***
E17	0.08 ± 0.02	17.76 ± 2.37	231.60 ± 45.53	44.67 ± 0.23 ***	177.92 ± 0.29 ***	75.18 ± 0.77 ***	1.26 ± 0.17 **
E36	0.14 ± 0.03 ***	20.09 ± 3.53 **	146.83 ± 15.50	39.94 ± 0.10 ***	159.32 ± 0.37 ***	67.97 ± 0.17 ***	0.94 ± 0.22 **
E37	0.15 ± 0.04 ***	13.12 ± 2.52	92.48 ± 17.82**	34.40 ± 0.03 ***	133.61 ± 0.64 ***	54.61 ± 0.64 ***	0.88 ± 0.17 ***
E42	0.10 ± 0.03	18.02 ± 3.11	188.57 ± 37.70	43.02 ± 0.54 ***	174.35 ± 1.62 ***	76.43 ± 0.83 ***	2.25 ± 1.04
E45	0.16 ± 0.02 ***	27.58 ± 4.78 ***	171.16 ± 26.94	36.61 ± 0.04 *	142.81 ± 0.36 ***	59.02 ± 0.35 **	2.24 ± 1.04
E53	0.07 ± 0.02	12.50 ± 2.19	210.79 ± 62.56	34.37 ± 0.06 ***	132.38 ± 0.10 ***	53.01 ± 0.26 ***	1.32 ± 0.28 **
E76	0.11 ± 0.01**	21.17 ± 2.25 **	210.79 ± 62.56	44.63 ± 0.12 ***	171.90 ± 0.06 ***	68.26 ± 0.37 ***	0.64 ± 0.01 ***
E107	0.07 ± 0.01	13.87 ± 2.95	194.36 ± 30.79	36.43 ± 0.04 **	134.20 ± 0.28 ***	48.49 ± 0.44 ***	0.42 ± 0.12 ***
IL12-4-SL	0.10 ± 0.02	21.00 ± 1.83 **	217.16 ± 48.16	39.71 ± 0.07 ***	149.58 ± 0.11 ***	56.68 ± 0.20 ***	2.77 ± 0.24 *
JAG8810	0.07 ± 0.01	13.46 ± 1.86	203.15 ± 55.39	42.96 ± 0.29	161.63 ± 1.51	60.49 ± 1.25	2.99 ± 0.5
LA2662	0.12 ± 0.01 **	21.59 ± 2.07 **	190.13 ± 24.57	36.98 ± 0.03 ***	143.90 ± 0.42 ***	58.78 ± 0.47 **	1.97 ± 0.66
LA3120	0.13 ± 0.03 *	20.57 ± 1.14 ***	166.53 ± 36.45	39.30 ± 0.13 ***	155.43 ± 0.23 ***	65.78 ± 0.60 ***	1.91 ± 0.98
M82	0.15 ± 0.03 ***	24.37 ± 4.93 ***	163.26 ± 12.14	32.87 ± 0.14 ***	121.82 ± 0.23 ***	44.88 ± 0.20 ***	3.25 ± 0.6 *

**Table 2:** Pearson’s correlations between physiological parameters, pigment content and crop yield production (* *p* < 0.05; ** *p* < 0.01). YP = yield *per* plant; F_v_/F_m_ = maximum quantum yield of PSII; Φ_PSII_ = effective quantum yield of PSII; NPQ = non-photochemical quenching; DW = leaf dry weight; SLA = specific leaf area; LA = leaf area; Chl a = chlorophyll a; Chl b = chlorophyll b; Car = carotenoids.

	YP	F_v_/F_m_	Φ_PSII_	NPQ	DW	SLA	LA	Chl a	Chl b	Car
YP	1	0.254	−0.010	−0.647 **	0.192	0.026	0.376	−0.017	0.036	−0.048
F_v_/F_m_		1	0.506	−0.266	0.304	−0.405	0.256	0.219	0.281	0.167
Φ_PSII_			1	−0.115	0.161	−0.124	0.002	−0.022	0.139	−0.147
NPQ				1	−0.068	−0.049	−0.183	0.106	0.035	0.154
DW					1	−0.650 **	0.744 **	0.155	0.221	0.110
SLA						1	−0.149	−0.226	−0.307	−0.167
LA							1	0.251	0.212	0.281
Chl a								1	0.970 **	0.983 **
Chl b									1	0.909 **
Car										1

**Table 3:** Heat-induced changes in chlorophyll fluorescence parameters characterizing the shape of slow Kautsky kinetics recorded for 8 tomato genotypes. Values are means of 5 replicates. Standard deviation (not shown here) was under 4% of means. Asterisks indicate the statistical significance of the difference in heat treated leaves compared to their respective non-treated control (* 0 to 50%; ** 50 to 100%; *** over 100%). O (origin) = minimum fluorescence level (also termed F_0_); P = peak fluorescence level reached after 1–2 s of actinic light exposure; S = semi-steady state of fluorescence emission; M = maximum of fluorescence; T = terminal steady state chlorophyll fluorescence of the slow Kautsky kinetics; Fp = fluorescence peak; Fs = fluorescence steady state; Rfd = relative fluorescence decline (vitality index); *t_P_* = time at which P fluorescence peak is reached; *t_S_* = time at which S fluorescence level is reached; *t_M_* = time at which M fluorescence level is reached; *t_T_* = time at which T fluorescence level is reached, Dip = decrease.

**Control**								
	**BG1620**	**E41**	**E107**	**IL12-4-SL**	**JAG8810**	**LA2662**	**LA3120**	**M82**
O/P	0.229	0.242	0.212	0.220	0.201	0.239	0.194	0.207
P/S	1.810	1.167	1.065	1.517	1.405	1.242	1.193	1.673
P/M	1.669	-	1.059	1.389	1.318	-	1.163	1.486
S/M	0.922	-	0.994	0.916	0.938	-	0.975	0.888
P/T (steady state)	2.665	3.101	2.101	2.482	2.428	2.887	2.694	2.801
M/T (steady state)	1.597	-	1.985	1.787	1.842	-	2.316	1.886
Rfd = (Fp−Fs)/Fs	1.665	2.101	1.101	1.482	1.428	1.887	1.694	1.801
*t*_P_	1.120	1.120	0.960	1.200	1.040	1.040	1.280	1.040
*t*_S_	6.080	3.360	4.080	4.080	4.080	4.080	−29.940	6.080
*t*_M_	10.080	-	2.720	10.080	8.080	-	6.080	12.080
*t*_T_	270.060	270.060	270.060	270.060	270.060	270.060	270.060	270.060
Dip at	26.080	-	52.080	42.080	-	48.080	86.080	38.080
**Heat Treated (60 min at 45 °C)**								
	**BG1620**	**E41**	**E107**	**IL12-4-SL**	**JAG8810**	**LA2662**	**LA3120**	**M82**
O/P	0.713 ***	0.483 **	0.341 **	0.344 **	0.366 **	0.252 *	0.379 **	0.316 **
P/S	-	-	-	1.280 *	0.936 *	-	-	0.855 *
P/M	-	-	-	1.166 *	0.929 *	-	-	0.853 *
S/M	-	-	-	0.911 *	0.993 *	-	-	0.997 *
P/T (steady state)	1.658 *	2.729 *	2.680 *	2.250 *	2.407 *	2.779 *	2.881 *	2.302 *
M/T (steady state)	-	-	-	1.929 *	2.591 *	-	-	2.700 *
Rfd = (Fp-Fs)/Fs	0.658 **	1.729 *	1.680 **	1.250 *	1.407 *	1.779 *	1.881 *	1.302 *
*t*_P_	1.280 *	1.280 *	1.760 **	1.120 *	1.280 *	1.040	1.280	1.040
*t*_S_	-	-	-	4.080	2.240 ***	-	-	3.360
*t*_M_	-	-	-	8.080	2.400 ***	-	-	4.080
*t*_T_	255.060 *	270.060 *	245.060 *	260.060 *	245.060 *	260.060 *	270.060 *	250.06 *
Dip at	-	-	-	34.080 *	40.080	-	-	40.08 *

**Table 4:** Tomato genotypes analyzed in this study.

No.	Genotype	Origin	Common Name
1	E7	Italy	Corbarino PC04
2	E8	Italy	Corbarino PC05
3	E17	Italy	Pantano Romanesco
4	E36	Italy	Riccia San Vito
5	E37	Italy	Siccagno
6	E41	Italy	Parmitanella
7	E42	Italy	PI15250
8	E45	Italy	SM246
9	E53	South America	Latin American cultivar (Honduras)
10	E76	URSS	Black Plum
11	E107	Europe	E-L-19, Spain
12	JAG8810	-	Monsanto F1 hybrid
13	M82	California	M82
14	IL12-4-SL	Italy	IL12-4-SL
15	LA2662	-	Saladette
16	LA3120	-	Malintka
17	BG1620	Bulgary	-
